# Neglected Tropical Diseases, Neglected Communities, and Conflict: How Do We Leave No One Behind?

**DOI:** 10.1016/j.pt.2017.10.013

**Published:** 2018-03

**Authors:** Julie Jacobson, Simon Bush

**Affiliations:** 1 Bill & Melinda Gates Foundation, 500 Fifth Avenue North, Seattle, WA 98109, USA; 2 Sightsavers, PO Box KIA 18190, Airport, Accra, Ghana

## Abstract

Most well established neglected tropical disease (NTD) programs have seen great progress towards disease control or elimination. Areas in conflict, however, are a looming challenge to reaching control and elimination targets. To be successful, programs and partners need to creatively adapt to local circumstances and embrace new colleagues not traditionally seen as NTD implementers.

To reach control and elimination targets for NTDs, inequity, which drives some populations to systematically not receive services and treatment, needs to be tackled. The biggest threat to elimination efforts is systematic noncompliance. This noncompliance is frequently misunderstood as refusal. The most common source of systematic noncompliance is, however, weak programs and lack of access to certain areas and communities. Inability to reach and treat in all endemic communities will mean that control and elimination targets will be difficult if not impossible to meet.

Mass drug administration (MDA) is designed to reach community members, wherever they live, with treatment and preventative services [1]. Similarly, test and treat programs, like those designed for human African trypanosomiasis (HAT), utilize mobile teams to reach people at risk in their villages. Both reach communities beyond the easy reach of health facilities.

To achieve elimination targets and equity, it is important to understand why we have not reached all communities. Frequently the stock answer is funding. Where this may be a factor, it is an oversimplification of a complex challenge. For instance, in countries that have yet to provide MDA for lymphatic filariasis (LF), there is a mix of frequently interdependent contributing factors: lack of data, poor political will, insecurity, and limited infrastructure. The combination of these factors leads to challenges in establishing and sustaining a program as well as raising resources from external sources, where donors may feel that the investment would not yield results and could be better spent elsewhere. Additionally, programs in these settings are frequently more expensive, requiring more resource-intensive approaches. Despite the challenges, there is progress being made, and in these very settings MDA can be a powerful tool, empowering communities to take part in their own struggle to improve health and development.

One of the reasons for lack of access is conflict and political instability (Figure 1). Conflict and NTDs are interrelated; circumstances encountered in conflict can both increase exposure and/or increase susceptibility to infection 1, 2. Below, we explore some examples of how this problem is being approached and the principles emerging from these programs, which are:

• adaptive and nimble programming;
• innovation, embracing new tools and approaches to deliver high-impact interventions in a shorter timeframe;
• flexible funding to allow adaptive programming and engaging new donors, including domestic resources;
• opportunistic partnerships and learning from organizations well-versed in working in conflict zones;
• empowering communities so that they can continue, even when external support is intermittent or unavailable.

**Figure 1:**
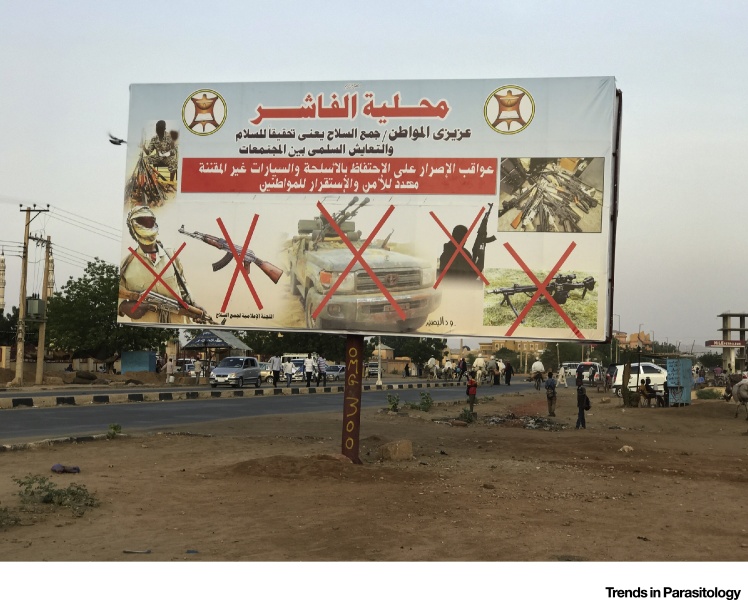
The Challenges of Control of Neglected Tropical Diseases in Conflict Areas. El Fashir, North Darfur State, Republic of the Sudan. Image credit of Simon Bush.

## Guinea Worm Eradication Program (GWEP): Opportunistic Partnering and Empowering Communities

The program that most clearly demonstrates the challenge of access to remote populations is the GWEP, with no drug, no diagnostic, and no vaccine to deploy. The program relies on identifying and treating Guinea worm (GW)-infected individuals and keeping them from contaminating additional water sources and spreading the infection. Conflict is playing a significant role in eradication, especially in the end game. The partners bring complementary skills to help: the Carter Center (www.cartercenter.org), working on promoting both peace and health, and the World Health Organisation (WHO), with their ability to reach displaced populations. The GWEP has had to evacuate their international staff multiple times due to conflict, and access to several areas is severely limited; they frequently maintain only local staff and community members. This challenge is most pronounced in South Sudan and Northern Mali. A strong grass roots approach, working with community volunteers as the basis of the surveillance and reporting system, enables the program to still function despite insecurity. Introducing rewards for confirmed cases means that everyone can be part of the surveillance system. The program also opportunistically utilizes other health programs, such as polio immunization campaigns, MDA, and Child Health Days as the eyes and ears of the program to identify suspected cases.

## South Sudan: Adaptive Programming and New Partners

Sightsavers (www.sightsavers.org) has supported the onchocerciasis program in Western Equatoria State, South Sudan, since 2014; however, in recent years geographic coverage has been compromised by worsening insecurity, and it fell from 462 000 treatments in 2015 to 196 000 treatments in 2016i. As a response, Sightsavers adapted the operational model of this project with the agreement of the donor, ministry, and implementing partners. The MENTOR Initiative (www.thementorinitiative.org), which has a strong track record of working in conflict zones, with access to most of the country, was subcontracted to undertake program implementation at community level with technical oversight remaining with Sightsavers disease experts. Although data are yet to be verified, it is expected that this change of approach will lead to 431 000 treatments being delivered in 2017 for onchocerciasis. In addition, the program will treat for LF in recently mapped areas that are coendemic with onchocerciasis. Security of the team remains a priority. Even with this approach, some areas are excluded from the project due to high insecurity. Security needs are continually reviewed by all partners in order to assess risk and to look for new opportunities to reach current ‘no-go areas’.

## Syria and Leishmaniasis: Nimble Programming, Opportunistic Partnering, and Empowering Communities

In northern Syria, the population is severely affected by conflict and an epidemic of leishmaniasis spread by the sandflies that breed exponentially in the debris and cracks of bombed-out buildings. This disease is the most common communicable disease across northern Syria, and in 2013 over 200 000 cases were reported [3]. Since mid-2013, the MENTOR Initiative has trained and operated a full team of professional and highly dedicated Syrian medical, prevention, surveillance, logistical, and administrative staff. These teams are supported, supplied, and managed by MENTOR teams on the Turkish and Iraqi borders. Local medical staff, including very basic health assistants from over 180 health facilities and mobile clinics, ensure access to effective diagnostic and treatment services protecting over 5 million vulnerable people using standardized case management protocols and supply-chain processes. In 2016, this impressive team was able to spray the homes of 3 475 575 people, and distribute 619 134 small-mesh insecticidal nets (bringing the total number distributed by the team since 2014 to around 2 million) [3]. Combined with targeted waste management, the use of treated curtain materials in urban settings, and sustained education through radio, mosques, schools, and communities, case numbers have decreased by 50% despite the escalating conflict [3].

## END Fund: Flexible Funding and Opportunistic Partnering

The ability to secure funding for programs in a conflict setting has been highlighted as a key challenge due to risk and higher costs. In addition, some donors may have restrictions on providing funding in conflict areas. The END Fund (www.end.org) has proven to be an important partner, being flexible and nimble with funding and able to adapt to the unique challenges seen in conflict. Since 2012 they have disbursed over 11 million US dollars to more than 10 countries in conflict situations and have seen impact reaching over 58 million beneficiaries with almost 107 million treatments (Table 1). The END Fund has targeted smaller areas where programs may be possible in Somalia, and it works through partners active in conflict settings such as the World Food Program in Afghanistan. In some places, limited strategic investments, such as technical assistance in Yemen, helped to release millions of dollars to support the NTD efforts from the World Bank, and strategic gap-filling in Mali allowed the NTD program to continue when USAID funding had to be suspended during a coup.

**Table 1:** The Reach of Neglected Tropical Disease (NTD) Programs in Countries in Conflict Supported by the END Fund, 2012–2018^a^

Country	Implementing partners	Years active	Beneficiaries^d^	Treatments	Workers trained
Afghanistan	World Food Programme *World Health Organisation*	2015–2017	7 200 000	7 200 000	
Angola^b^	MENTOR Initiative	2013–2018	*3* *884* *645*	*8* *789* *429*	*32* *008*
Central African Republic (CAR)	CBM Organisation pour la Prévention de la Cécité World Health Organisation	2015–2017	2 750 625	3 503 591	11 643
Mali^c^	Helen Keller International	2012–2018	21 972 618	46 344 593	46 780
Somalia	World Health Organisation	2016–2017	*897* *825*	*1* *778* *807*	
South Sudan	Sightsavers World Food Programme	2015–2018	*1* *465* *944*	*1* *465* *944*	*5364*

a www.end.org/ourimpact/annual-reports-and-financial-statements/5-year-impact-report.

b This project also conducted mapping.

c This project also provided surgeries for lymphatic filariasis.

d *Italics* denotes work in progress.

## New Tools, New Approaches, New Hope

To be successful, MDA programs rely on consistent annual access with good coverage over many years. New, more effective, treatments, such as the combination of ivermectin, diethylcarbamazine (DEC), and albendazole (IDA triple-drug) for LF, may be more effective when sustained access is not possible. With efficacy superior to the standard two-drug treatment of ivermectin and albendazole, IDA triple-drug may require only one or two high-coverage-quality treatment years, instead of 5–7 years or more [4]. This could be a critical tool to be successful in areas of conflict where access is limited. Similarly, fexinidazole for HAT, could be transformative as an oral treatment that can be provided to the community and does not require intravenous infusions or invasive lumbar puncture – which required trained staff and a health care facility [5]. Additionally, control methods that are highly effective against HAT vectors, and use a new technology based on an insecticide-treated screen ('tiny targets') to control flies more effectively [6], can be easily deployed by communities to further decrease the parasite pool and transmission [7]. New, more effective, treatments – like the combination of IDA triple-drug for LF, or fexinidazole for HAT – may be key to success in areas where access is limited or intermittent.

## Conclusion

With alternative flexible and adaptive approaches, embracing new partners, and empowering communities, we can expand more effectively and efficiently to achieve the control and elimination goals of the NTD programs globally. Innovations in MDA approach and new tools may be critical to success in these most difficult-to-reach populations. Learning from the approaches discussed here will help to make progress, but additional lessons and innovation will be needed to ultimately achieve elimination.

One thing is clear, these challenges must be overcome to achieve the NTD goals as well as health and development goals more broadly. Successful NTD programs are an indicator of initial health service access in hard-to-reach areas. Hopefully, they will help to pave the way to more resilient communities, higher demand from individuals, and expanded reach of programs for health and development services in these settings.

## Resources

^i^www.sightsavers.org/where-we-work/south-sudan/
